# SpuA-Mediated Glycogen Metabolism Modulates Acid Stress Adaptation via Formic Acid and Amino Acid Utilization in *Streptococcus pneumoniae*

**DOI:** 10.3390/microorganisms13102409

**Published:** 2025-10-21

**Authors:** Weichen Gong, Masayuki Ono, Xuefei Cheng, Yujiro Hirose, Keita Nishiyama, Haruki Kitazawa, Shigetada Kawabata

**Affiliations:** 1 Laboratory of Animal Food Function, Graduate School of Agricultural Science, Tohoku University, Sendai 980-8572, Japan; 2 Department of Microbiology, Graduate School of Dentistry, Osaka University, Osaka 565-0871, Japan; 3 Livestock Immunology Unit, International Education and Research Center for Food and Agricultural Immunology (CFAI), Graduate School of Agricultural Science, Tohoku University, Sendai 980-8572, Japan; 4 Department of Microbiology and Immunology, Nihon University School of Dentistry at Matsudo, Chiba 271-8587, Japan

**Keywords:** glycogen utilization, formic acid, adaptation, amino acid utilization, serum amyloid A1

## Abstract

Glycogen metabolism plays a key role in bacterial adaptation. In *Streptococcus pneumoniae*, the glycogen-degrading enzyme SpuA is widely conserved, but its physiological significance remains unclear. In this study, we investigated how SpuA affects bacterial growth and response to acid stress. We found that the *spuA* deletion strain (Δ*spuA*) produced more acidic metabolites under anaerobic conditions than the wild-type strain. In a mouse infection model, bronchoalveolar lavage fluid (BALF) from Δ*spuA*-infected mice was more acidic on day 1 post-infection, showing a lower bacterial load than wild-type infection—a finding consistent with the early growth delay observed in vitro—but the mutant later exhibited enhanced persistence at 72 h. Δ*spuA* strains also showed greater tolerance to formic acid and higher intake of serum amyloid A1 (SAA1), which may further contribute to their survival in acidic environments. Transcriptomic analysis revealed reduced utilization of certain amino acids, particularly cysteine, in Δ*spuA* strains. However, the addition of 0.05% (*v*/*v*) formic acid restored amino acid utilization in Δ*spuA* strains, and co-supplementation with formic acid and cysteine significantly enhanced Δ*spuA* growth in vitro. These findings suggest that in the absence of SpuA, *S. pneumoniae* shifts its metabolism toward formic acid production, which may act both as a metabolic signal and a stressor that influences bacterial gene expression. This shift is accompanied by increased expression of tRNAs and growth rescue, suggesting enhanced amino acid utilization capacity. Although our findings reveal a potential link between formic acid metabolism and amino acid utilization through tRNA regulation, further validation using metabolic flux analyses or targeted metabolomics will be required to confirm this relationship. These observations imply a metabolic adaptation that facilitates bacterial growth under low-oxygen, acidic conditions during infection. Our results also raise the possibility that SpuA plays a role in restraining bacterial overgrowth in the host, thereby promoting a more balanced coexistence between pathogen and host.

## 1. Importance

*S. pneumoniae* is a major respiratory pathogen that adapts to diverse environments during infection. While SpuA, a glycogen-degrading enzyme, is widely conserved in *S. pneumoniae*, its biological role has remained unclear. Here, we show that loss of SpuA leads to increased acid production, enhanced tolerance to acidic stress, and accelerated bacterial proliferation during infection. These changes are driven by metabolic shifts toward formic acid production and improved amino acid utilization, particularly under low-oxygen conditions. Our findings reveal a previously unrecognized link between glycogen metabolism and acid stress adaptation in *S. pneumoniae*. Importantly, we propose that SpuA may act as a metabolic checkpoint that limits excessive bacterial growth in the host, supporting a more stable host–pathogen balance. Understanding this regulation may offer new strategies for controlling pneumococcal colonization and persistence.

## 2. Introduction

Carbohydrates are a preferred energy source for many organisms, providing ATP primarily through glycolysis and the tricarboxylic acid cycle. *Streptococcus pneumoniae* (*S. pneumoniae*), a facultative anaerobic Gram-positive bacterium, relies heavily on carbohydrate metabolism to support its growth, colonization, and pathogenicity [1]. In host environments, however, free glucose levels are often limited. Instead, carbohydrates are predominantly stored as glycogen, a highly branched glucan, mainly in the liver and skeletal muscles [2]. Recent studies have shown that glycogen is also present in nontraditional storage sites, including the lungs [3]. Glycogen accumulation has been observed in respiratory epithelial cells and alveolar type II cells, particularly during embryogenesis or under stress conditions such as hypoxia or infection [4]. These glycogen reserves are thought to supply metabolic support when local energy is scarce. Moreover, partial degradation of glycogen may release monosaccharides that serve as carbon sources for colonizing microbes, including pathogens like *S. pneumoniae* [5].

*spuA* in *S. pneumoniae* encodes SpuA, an extracellular pullulanase that degrades alpha glucans such as glycogen. SpuA is considered an important virulence factor because it shows strong affinity for glycogen in alveolar type II epithelial cells, promoting bacterial adherence and colonization [6]. Similar enzymes have been identified in other streptococcal species, such as *Streptococcus suis*, where SpuA-like proteins also contribute to pathogenicity. The ApuA protein (a SpuA homolog) in *S. suis*, for example, is anchored to the cell surface and can cleave both α-1,4 and α-1,6 glycosidic bonds, facilitating the degradation of extracellular polysaccharides [7]. In *S. pneumoniae*, the expression of *spuA* is upregulated in response to glycogen and supports bacterial attachment to host cells [8].

While the role of *spuA* has been studied in the D39 strain of *S. pneumoniae* (serotype 2), the deletion of *spuA* resulted in a strain with reduced competitiveness in a mouse model of virulence relative to the parent strain, linking the degradation of host-glycogen to the virulence of the bacterium [6]. However, the function of *spuA* in the TIGR4 strain (serotype 4) remains unclear. In this study, we constructed a *spuA*-deficient mutant in the TIGR4 background and evaluated how the loss of *spuA* affects bacterial metabolism and colonization. By combining in vitro culture assays with a mouse nasal infection model, we identified phenotypic differences from previous observations in the D39 strain.

This study aims to determine whether alveolar glycogen serves as a key nutrient source for *S. pneumoniae*, and to explore how deletion of *spuA* influences colonization ability and metabolic adaptation in the host lung environment.

## 3. Materials and Methods

Our research complied with all of the relevant ethical regulations. All of the animal procedures were conducted according to the protocols approved by Animal Care and Use Committee of Osaka University Graduate School of Dentistry (authorization number 04-018-0).

All methods are reported in accordance with the ARRIVE guidelines (Animal Re-search: Reporting of In Vivo Experiments) to ensure rigorous and transparent reporting of animal research (https://arriveguidelines.org (accessed on 12 February 2025)).

### 3.1. Bacterial Culture

*Streptococcus pneumoniae* TIGR4 (virulent serotype 4 clinical isolate), purchased from commercial suppliers, was cultured in Todd-Hewitt broth (Becton, Dickinson and Company [BD], Franklin Lakes, NJ, USA) supplemented with 0.2% yeast extract (BD) (THY broth) at 37 °C in 5% CO_2_ incubator.

### 3.2. Construction of ΔspuA Mutant Strain

*S. pneumoniae* TIGR4 isogenic Δ*spuA* mutant strain was generated as previously described with minor modifications [9,10,11]. Briefly, the upstream region of *spuA*, an *aad9* cassette, and the downstream region of *spuA* were amplified using PrimeSTAR MAX DNA Polymerase (TaKaRa Bio, Shiga, Japan) and the specific primers listed in Supplementary Table S1. The DNA fragments were assembled using overlap extension PCR, which is achieved via two-step reactions: the first with three fragments and no primers for 15 cycles, and the second using primers binding to both ends of the DNA for 30 cycles. The assembled linear DNA was then used to construct mutant strains by double-crossover recombination with the synthesized competence-stimulating peptide-2 (GLPBIO, Montclair, CA, USA). The mutation was confirmed by Sanger sequencing.

### 3.3. Animal Infection Experiments

Male Slc:ICR mice at 6 to 7 weeks old (SLC Japan, Inc., Shizuoka, Japan) were used as animal infection model in this study. The *S. pneumoniae* TIGR4 wild-type and Δ*spuA* strains were grown to the early-exponential phase (The culture was initiated at an OD_600_ of 0.003 and incubated at 37 °C for 4 h, reaching an OD_600_ of 0.1.) and then washed with and resuspended in PBS.

For the experiments shown in Figure 2, three independent infection experiments were performed. In each experiment, 14 mice were infected with wild-type and 14 mice with Δ*spuA* strains. At 24 h post-infection, 7 mice from each group were euthanized, and BALF was collected from each mouse to measure bacterial load, pH, and SAA levels. At 72 h post-infection, the remaining mice in each group were euthanized, and BALF was collected to assess bacterial load, pH, and SAA levels.

For intranasal infection experiments, each mouse was intraperitoneally given 250 µL of anesthetic consisting of 20 µL midazolam (Takeda Pharmaceuticals, Osaka, Japan), 7.5 µL domitor (ZENOAQ, Fukushima, Japan), 25 µL vetorphale (Meiji Animal Health Co., Ltd., Kumamoto, Japan) and 197.5 µL PBS solution, then bacteria were given to mice by administration of 4 × 10^6^ CFU in 20 µL PBS. BALF were collected from mice 24 h and 72 h post intranasal infection after mice were euthanized by intraperitoneal injection of 300 µL pentobarbital sodium solution (20 mg/mL, Product number: P0776, Tokyo Chemical Industry Co., Ltd., Tokyo, Japan). BALF were collected by slowly drawing the injected PBS (1 mL) back into the syringe and serum was obtained from blood by centrifugation (8500 rpm, 15 min, 4 °C). The collected BALF was defined as the original (undiluted) concentration, and serial dilutions were subsequently performed for bacterial load detection, pH and SAA1 level measurement.

### 3.4. Quantification of SAA1 Using ELISA

After the collection of BALF from mice, SAA1 quantification was performed by ELISA following the manufacturer’s protocols described in mouse serum amyloid A ELISA Kit (SAA; Cat# KMA0021, Invitrogen, Thermo Fisher Scientific, Waltham, MA, USA).

### 3.5. Incubation of S. pneumoniae with THY Broth Containing 20% Serum In Vitro

Serum was collected from Slc:ICR mice at 6 to 7 weeks old (SLC Japan, Inc., Shizuoka, Japan) and the SAA1 concentration was measured by mouse serum amyloid A ELISA Kit (SAA; Cat# KMA0021, Invitrogen, Thermo Fisher Scientific, Waltham, MA, USA).

*S. pneumoniae* TIGR4 strain was grown to the early-exponential phase (OD_600_ of 0.1). Then, 5 µL of bacterial culture was added to 20 µL serum and 175 µL THY broth (containing final 0.05% formic acid, *v*/*v*) in a 96-well plate and incubated in 5% CO_2_ incubator for 12 h. After incubation, the supernatant of bacterial culture was collected after centrifugation (12,000 rpm, 10 min, 4 °C) and the remaining SAA was measured using mouse serum amyloid A ELISA Kit (SAA; Cat# KMA0021, Invitrogen, Thermo Fisher Scientific, Waltham, MA, USA) [12].

### 3.6. pH Measurement of Bacterial Culture Under Aerobic and Anaerobic Environments

A 500 µL volume of wild-type and Δ*spuA S. pneumoniae* TIGR4 strains (OD_600_ = 0.003) was added to each well of 24-well plate, then cultured in aerobic (5% CO_2_ incubator) and anaerobic environments created by using AnaeroPack-Anaero (MITSUBISHI GAS CHEMICAL Co. Inc., Tokyo, Japan). The supernatant of bacterial cultures was collected at different time points (1, 3, 5, 7, 9 h). The pH of all bacterial cultures was measured at room temperature using a HORIBA LAQUAtwin-pH-11 portable pH meter (HORIBA, Kyoto, Japan) following the manufacturer’s instructions.

### 3.7. Bacterial Growth Curve and Doubling Time Calculation

Wild-type and Δ*spuA S. pneumoniae* TIGR4 strains were grown to the early-exponential phase (OD_600_ of 0.1). Then, 5 µL of the bacterial culture was added to 195 µL of THY solution supplemented with either 0.075% (*v*/*v*) acetic acid, 0.075% (*v*/*v*) formic acid, or various amino acids (25 µM cysteine, alanine, serine, glutamic acid or glutamine), depending on the experimental condition. The doubling time was calculated using the OD_600_ values measured at the beginning and end of a 3 h window during the logarithmic growth phase. The values were entered into the online doubling time calculator provided by Omni Calculator (https://www.omnicalculator.com/math/doubling-time (accessed on 12 February 2025)).

### 3.8. RNA Extraction and RNA Sequencing

*S.pneumoniae* TIGR4 wild-type and Δ*spuA* strains were cultured in THY medium to early-exponential phase (OD_600_ ≈ 0.2) in the presence or absence of 0.05% (*v*/*v*) formic acid, depending on the experimental condition. A total of 1.5 mL of each culture was harvested by centrifugation at 6000× *g* for 10 min at 4 °C. The bacterial pellets were resuspended in 600 µL of lysis buffer (provided in the RNeasy Mini Kit #74104, Qiagen, Hilden, Germany) and subjected to mechanical disruption in Lysing Matrix B using a MagNA Lyser (Roche, Basel, Switzerland).

Following cell lysis, total RNA was extracted using the RNeasy Mini Kit (#74104, Qiagen, Hilden, Germany) according to the manufacturer’s protocol, including on-column DNase I treatment to remove residual genomic DNA. RNA concentration and purity were assessed using a NanoDrop One Microvolume UV-Vis Spectrophotometer (Thermo Fisher Scientific, Waltham, MA, USA), and RNA integrity was evaluated by agarose gel electrophoresis. Only samples with A_260_/A_280_ ratios between 1.8 and 2.0 and clear 16S/23S rRNA bands were used for downstream analysis performed at the Genome Information Research Center, Research Institute for Microbial Diseases, The University of Osaka, Osaka, Japan. Full-length cDNA was generated using the SMART-Seq^®^ HT Kit (Takara Bio), according to the manufacturer’s instructions. Pair-end libraries were generated using a Nextera XT DNA Kit and sequenced using a NovaSeq 6000 system (both from Illumina, San Diego, CA, USA). RNA-seq data have been registered in the NCBI BioProject database (accession ID: PRJNA1289369).

### 3.9. Transcriptomic Analysis

Sequenced data were processed as previously described, with minor modifications [13]. Briefly, reprocessing was achieved using Trimmomatic v.0.33 and FastQC v.0.12.1. The reads were mapped to the complete TIGR4 genome (NCBI Refseq assembly: GCF_000006885.1) using STAR v.2.7.0a. After a second quality check using FastQC, read counting was performed using featureCounts v.1.5.2 [14]. Differentially expressed genes were identified using iDEP v.0.96, with statistical significance assessed based on an adjusted *p*-value (Benjamini–Hochberg correction) of <0.05 [15]. Plots were created using iDEP and the R package ggplot2.

## 4. Results

### 4.1. ΔspuA S. Pneumoniae Produces More Acidic Byproducts Under Low-Oxygen Conditions and Shows Increased Resistance to Formic Acid

**Figure 1:**
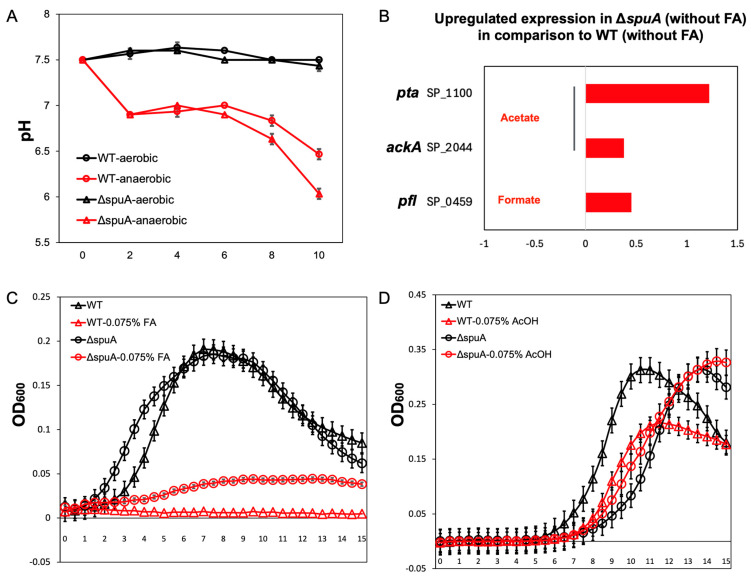
Δ*spuA S. pneumoniae* produced more acidic metabolites and showed enhanced tolerance to formic acid in anaerobic conditions. (**A**) Δ*spuA S. pneumoniae* produces more acidic metabolites in anaerobic conditions in comparison to those in aerobic conditions. *n* = 4 biological replicates per group. Data are presented as mean ± SD. (**B**) The genes expression relating to acetate (*pta* and *ackA*) and formate (*pfl*) secretion in Δ*spuA S. pneumoniae* were upregulated in Δ*spuA S. pneumoniae*. FA, formic acid. (**C**) Δ*spuA S. pneumoniae* showed enhanced tolerance to formic acid in comparison to wild-type strain. FA, formic acid. Data are presented as mean ± SEM. *n* = 3 biological replicates per group. (**D**) Under acetic acid stress, the growth of the Δ*spuA* strain and wild-type *S. pneumoniae* showed no significant difference from 0 to 11 h, but after 11 h, the growth of the Δ*spuA* strain was significantly enhanced. Data are presented as mean ± SEM. *n* = 3 biological replicates per group.

The respiratory pathogen *S. pneumoniae* can cause inflammation in the lungs and increase capillary leakage during infections in the lower respiratory tract. This leads to fluid accumulation in the lungs and reduces oxygen availability [16]. In response to this low-oxygen environment, *S. pneumoniae* shifts its metabolism toward anaerobic pathways [17]. Interestingly, the Δ*spuA* strain produced acidic byproducts more quickly than the wild-type (WT) strain under anaerobic conditions (Figure 1A). RNA sequencing showed that this increase in acidity was due to higher expression of genes involved in acetate (*pta*, *ackA*) and formate (*pfl*) production (Figure 1B). Since acidic conditions can be harmful to *S. pneumoniae*, the increased acid production by the Δ*spuA* strain was opposite to our expectations. Further tests revealed that the Δ*spuA* strain had much better survival in the presence of formic acid. While the WT strain could not grow at a concentration of 0.075% (*v*/*v*) formic acid, the Δ*spuA* strain was still able to grow (Figure 1C). Similarly, under acetic acid stress, the growth of the Δ*spuA* and wild-type strains was comparable during the first 11 h, but after 11 h, the Δ*spuA* strain exhibited significantly enhanced growth. (Figure 1D).

These results suggest that SpuA is closely connected to formic acid metabolism. The deletion of *spuA* leads to both increased formic acid production in low-oxygen conditions and improved resistance to formic acid.

### 4.2. ΔspuA S. Pneumoniae Shows Increased Growth Between 24 and 72 h After Infection in Mice

**Figure 2:**
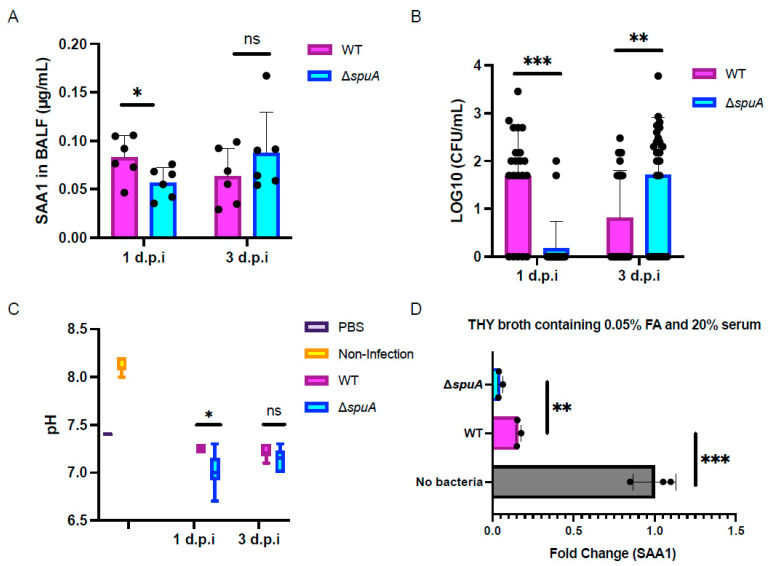
The production of acidic metabolites and stronger ability to intake SAA1 likely promote the growth of Δ*spuA S. pneumoniae.* (**A**) pH of BALF collected at 24 and 72 h after infection with either WT or Δ*spuA* strain. d.p.i, day(s) post-infection. Data are presented as mean ± SD. *n* = 7 biological replicates per group. ns, no significant difference; *, *p* < 0.05. Student’s *t*-test used for statistical analysis. The experiment was independently repeated three times with similar trends observed. The data presented are from one representative experiment. (**B**) Bacteria load in BALF collected at 24 and 72 h after infection with either WT or Δ*spuA* strains. Data are presented as mean ± SD. **, *p* < 0.01; ***, *p* < 0.005. Student’s *t*-test used for statistical analysis. *n* = 21 biological replicates per group. (**C**) SAA1 levels in BALF collected at 24 and 72 h after infection with either WT or Δ*spuA* strains. Data are presented as mean ± SD. *n* = 7 biological replicates per group; ns, no significant difference; *, *p* < 0.05. Student’s *t*-test used for statistical analysis. The experiment was independently repeated three times with similar trends observed. The data presented are from one representative experiment. (**D**) In vitro experiments revealed that Δ*spuA* strain showed stronger ability to intake SAA1 in 20% serum-THY broth. Data are presented as mean ± SD. *n* = 3 biological replicates per group. **, *p* < 0.01; ***, *p* < 0.005. Student’s *t*-test used for statistical analysis. The experiment was independently repeated three times with similar trends observed. The data presented are from one representative experiment.

As shown in vitro, the Δ*spuA* strain produces more acidic compounds under low-oxygen conditions (Figure 1A). To test if this also happens in living organisms, we carried out mouse infection experiments. Bronchoalveolar lavage fluid (BALF) was collected at 24 and 72 h after infection with either WT or Δ*spuA* strains. We measured both pH and bacterial load in the BALF. At 24 h after infection, BALF from the Δ*spuA*-infected group was more acidic than that from the WT-infected group, but the pH difference disappeared by 72 h (Figure 2A). Bacterial counts showed that fewer Δ*spuA* bacteria were present at 24 h, but significantly more were found at 72 h, suggesting rapid growth during this period from 24 h to 72 h post-infection (Figure 2B).

Previous studies have shown that *S. pneumoniae* can adapt to formic acid stress by internalizing serum amyloid A1 (SAA1) [12]. To explore whether the Δ*spuA* strain behaves similarly, we measured SAA1 levels in BALF using ELISA. The results showed lower SAA1 levels at 24 h in the Δ*spuA*-infected group (Figure 2C). To confirm whether this was due to increased internalization by bacteria, we performed co-culture experiments in vitro and again measured SAA1 using ELISA. The Δ*spuA* strain internalized more SAA1 than the WT strain (Figure 2D).

These results suggest that the Δ*spuA* strain has a stronger ability to take in SAA1, which may help it survive in acidic conditions and promote its growth during infection.

### 4.3. Formic Acid and Cysteine Promote the Growth of the ΔspuA S. pneumoniae

**Figure 3:**
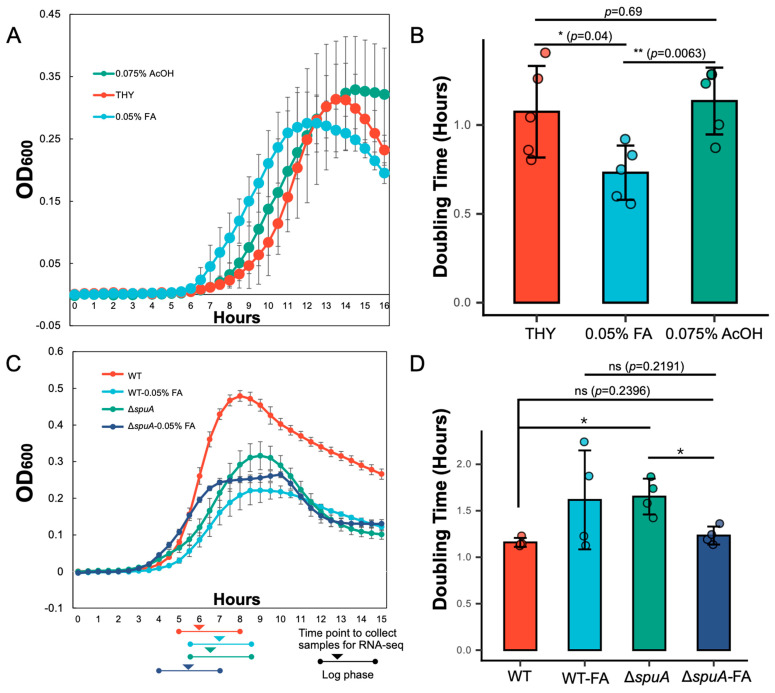
**Formic acid promotes the growth of the Δ*spuA S. pneumoniae*.** (**A**) Growth curve of Δ*spuA S. pneumoniae* in THY broth with addition of 0.075% (*v*/*v*) acetic acid and 0.05% (*v*/*v*) formic acid. *n* = 5 biological replicates per group. Data are presented as mean ± SEM. (**B**) Doubling time of Δ*spuA S. pneumoniae* in THY broth with addition of 0.075% (*v*/*v*) acetic acid and 0.05% (*v*/*v*) formic acid. *, *p* < 0.05; **, *p* < 0.01. Student’s *t*-test used for statistical analysis. *n* = 5 biological replicates per group. Data are presented as mean ± SD. (**C**) Growth curve of wild-type or Δ*spuA S. pneumoniae* in THY broth with/without 0.05% FA. *n* = 3 biological replicates per group. Data are presented as mean ± SEM. Samples for RNA sequencing were collected in middle log phase as indicated by arrow marker. (**D**) Doubling time of wild-type or Δ*spuA S. pneumoniae* in THY broth with/without 0.05% FA. ns, no significant difference; *, *p* < 0.05. Student’s *t*-test used for statistical analysis. *n* = 4 biological replicates per group. Data are presented as mean ± SD.

Based on the results from the animal infection experiments, we suspected that the acidic environment might support the increased growth of the Δ*spuA* strain (Figure 2A,B). We also observed that this strain produces more formic and acetic acids under low-oxygen conditions (Figure 1B). To test whether these acids affect bacterial growth, we added 0.075% acetic acid or 0.05% formic acid to THY broth and measured the doubling time of the Δ*spuA* strain. The results showed that 0.05% formic acid significantly improved its growth (Figure 3A,B). Without this addition, the Δ*spuA* strain grew more slowly than the wild-type strain, but with formic acid, its growth was noticeably faster (Figure 3C,D).

**Figure 4:**
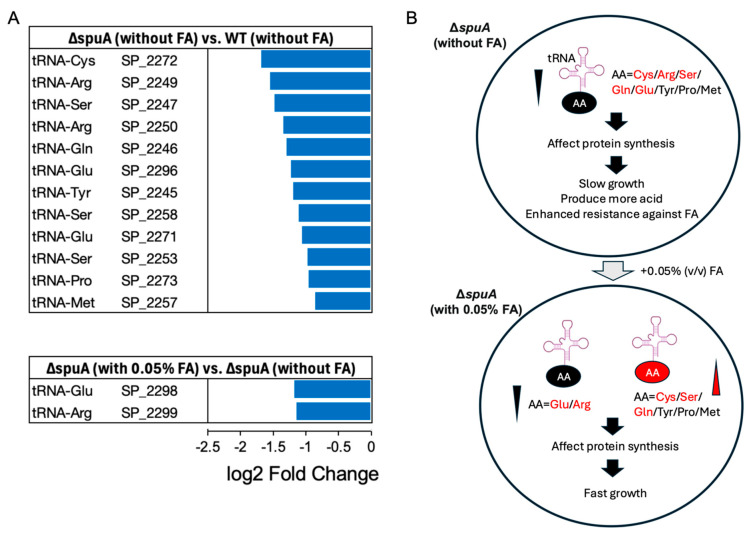
**The addition of formic acid restored the expression of tRNA relating to amino acid usage.** (**A**) RNA-seq results showed that the significant expression change (*p* < 0.05) of tRNA relating to multiple amino acid usage is downregulated in Δ*spuA S. pneumoniae.* Additionally, the addition of 0.05% (*v*/*v*) formic acid could restore the expression of most tRNAs relating to amino acid usage. *n* = 3 biological replicates per group. Data are presented as mean. (**B**) Graphic illustration of the change in tRNA expression relating to amino acid usage in Δ*spuA S. pneumoniae* with/without formic acid.

To explore how formic acid enhances growth, we performed RNA sequencing on WT and Δ*spuA* strains in mid-log phase, with and without 0.05% formic acid in the medium (Figure 3C). The data showed that in the absence of formic acid, the Δ*spuA* strain had reduced expression of tRNAs related to the transport of several amino acids, including cysteine (Cys), arginine (Arg), serine (Ser), glutamine (Gln), glutamic acid (Glu), tyrosine (Tyr), proline (Pro), and methionine (Met). When formic acid was present, the expression of most of these tRNAs increased, except those for glutamic acid and arginine (Figure 4A,B).

**Figure 5:**
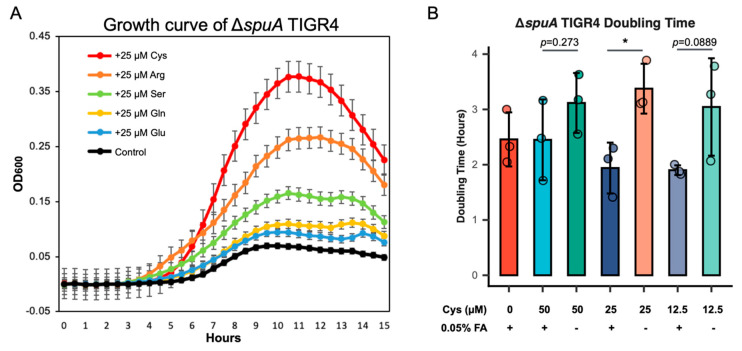
**The recovery of cysteine usage is important in Δ*spuA S. pneumoniae* growth.** (**A**) Growth curve of Δ*spuA S. pneumoniae* in 0.05% FA-THY broth with 25 µM of different amino acids, including cysteine (Cys), arginine (Arg), serine (Ser), glutamine (Gln) and glutamic acid (Glu). *n* = 3 biological replicates per group. Data are presented as mean ± SEM. (**B**) The combination of 12.5 µM, 25 µM and 50 µM cysteine with or without 0.05% (*v*/*v*) formic acid contributes to Δ*spuA S. pneumoniae* recovered growth. *, *p* < 0.05. *n* = 3 biological replicates per group. Data are presented as mean ± SD.

To identify which amino acid had the greatest effect, we added 25 µM of cysteine, arginine, serine, glutamine, or glutamic acid to THY broth containing 0.05% formic acid. Among them, only cysteine significantly boosted the growth of the Δ*spuA* strain (Figure 5A,B).

These findings suggest that the slower growth of the Δ*spuA* strain without formic acid is mainly due to limited use of key amino acids, especially cysteine. Supplementing with 0.05% formic acid, and particularly with both formic acid and cysteine, greatly improves the growth of the Δ*spuA* strain, making it comparable to the WT strain.

### 4.4. Comprehensive Analysis of Amino Acid Metabolism in the ΔspuA S. pneumoniae

The slower growth of the Δ*spuA* strain in the absence of formic acid appears to result from reduced use of certain amino acids. The supplementation of 0.05% (*v*/*v*) formic acid improved growth and restored amino acid utilization, except for glutamate and arginine (Figure 4A,B). Based on this observation, we hypothesized that when *S. pneumoniae* loses the ability to metabolize glycogen, it may shift toward using amino acids as its main energy source.

**Figure 6:**
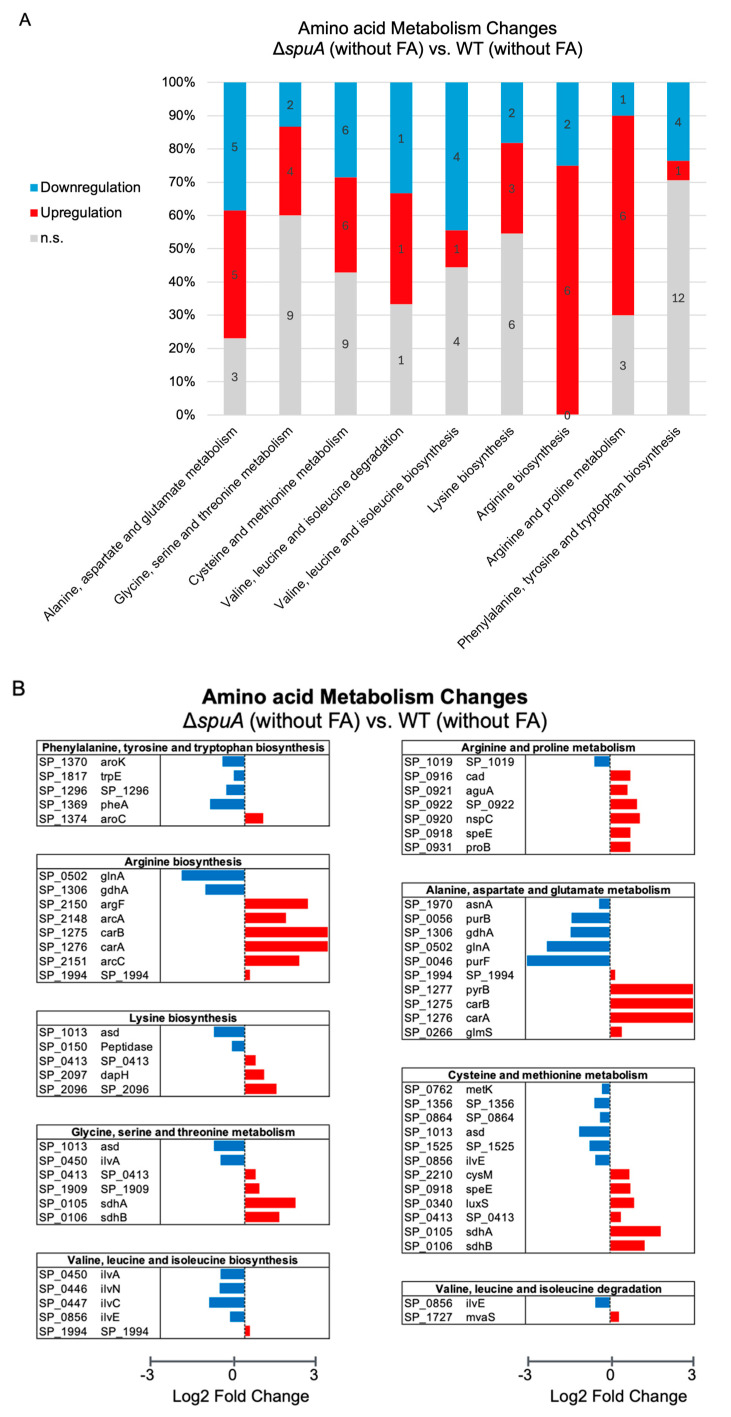

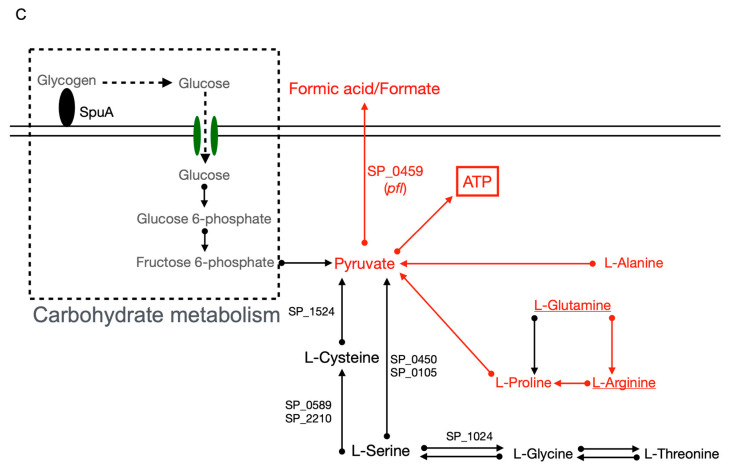
**Comprehensive analysis of amino acid metabolism in the Δ*spuA S. pneumoniae.*** (**A**) Amino acid metabolism changes between Δ*spuA* and wild-type *S. pneumoniae* using KEGG-based gene function annotations from the TIGR4 genome. Downregulation (blue) or upregulation (red) means those genes were significantly lower or higher expression in Δ*spuA* strain compared with wild-type strain. (**B**) Graphic summary of amino acid pathway associated with formate production in *S. pneumoniae* TIGR4 strain based on KEGG metabolism database. (**C**) Detailed information of amino acid metabolism changes between Δ*spuA* strain with or without the addition of 0.05% (*v*/*v*) formic acid. Genes were functionally categorized based on the KEGG metabolism database.

To explore this possibility, we analyzed the RNA sequencing data of the Δ*spuA* strain using KEGG-based gene function annotations from the TIGR4 genome. This in silico analysis revealed that 75% (6 out of 8) of genes associated with arginine biosynthesis were upregulated. Similarly, 60% (6 out of 10) of genes involved in arginine and proline metabolism, and 38.5% (5 out of 13) of genes related to alanine, aspartate, and glutamate metabolism showed increased expression (Figure 6A,B).

Additionally, the *pfl* gene (*SP_0459*), which encodes a key enzyme for formic acid production, was upregulated in the Δ*spuA* strain (Figure 1B). According to KEGG pathway maps, the metabolism of alanine, arginine, and proline can generate pyruvate, which serves as a precursor for formic acid production. These transcriptomic and pathway-based observations support the idea that the Δ*spuA* strain may compensate for impaired glycogen use by upregulating amino acid metabolic pathways that ultimately enhance formic acid production (Figure 6C).

**Figure 7:**
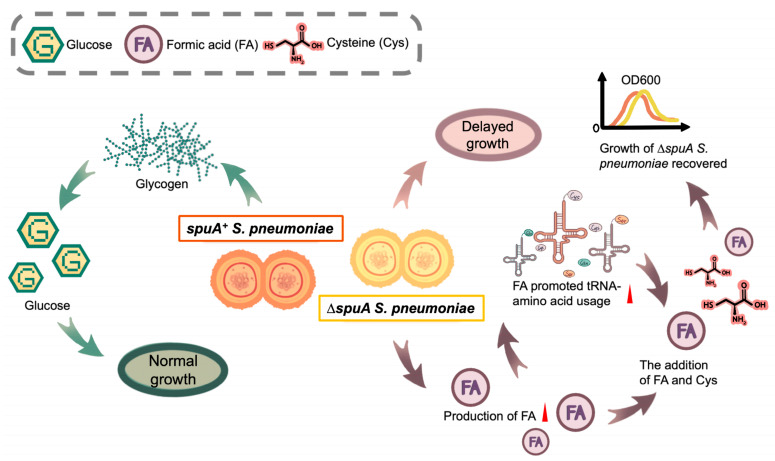
**The role of SpuA in mediating glycogen metabolism, bacterial growth and acid stress adaptation.** The absence of SpuA results in less efficient glycogen utilization and slower bacterial growth. To compensate, the Δ*spuA* strain shifts its metabolism toward increased formic acid production, which in turn facilitates amino acid utilization, particularly cysteine, and supports bacterial growth.

In conclusion, the absence of SpuA results in less efficient glycogen utilization and slower bacterial growth. To compensate, the Δ*spuA* strain shifts its metabolism toward increased formic acid production, which in turn facilitates amino acid utilization, particularly cysteine, and supports bacterial growth (Figure 7).

## 5. Discussion

This study reveals an unexpected role for SpuA in the metabolic adaptation and proliferation dynamics of *S. pneumoniae*. While SpuA has been traditionally regarded as a virulence factor that promotes colonization by degrading host glycogen, our findings suggest that its absence triggers compensatory metabolic shifts that support bacterial survival and proliferation, particularly under stress conditions such as acid exposure and limited nutrient availability.

The deletion of *spuA* impaired the bacterium’s ability to utilize α-glucans like glycogen, a key carbohydrate reservoir in alveolar cells [6]. As a consequence, *S. pneumoniae* lacking SpuA exhibited increased production of acidic metabolites, notably formic acid, under anaerobic conditions. This shift suggests a redirection of metabolic flux toward fermentative pathways, likely as a strategy to generate ATP in the absence of glycogen-derived substrates [18]. Concomitantly, the Δ*spuA* strain demonstrated enhanced tolerance to formic acid, indicating that the bacterium not only increased acid output but also adapted to the resulting intracellular stress.

In a murine *S. pneumoniae* infection model, we observed that Δ*spuA* bacteria initially exhibited attenuated colonization at 24 h post-infection. However, they showed a marked proliferation from 24 to 72 h post-infection, a pattern not previously reported in the well-characterized D39 strain. It is also worth noting that Δ*spuA* exhibited a lower bacterial load at 24 h post-infection compared with the wild-type strain, consistent with its initial growth delay observed in vitro. This suggests that *spuA* deletion may transiently impair early colonization or adaptation within the host environment. The subsequent increase in bacterial numbers at 72 h likely reflects compensatory metabolic adaptations, including improved tolerance to formic acid accumulation and altered amino acid utilization. This delayed yet significant increase in bacterial burden implies that the absence of SpuA leads to a temporary growth defect that is later compensated by alternative metabolic routes. The ability of the mutant to rebound in vivo suggests a high degree of metabolic flexibility in *S. pneumoniae*. However, it is also possible that factors beyond metabolic adaptation contribute to this delayed yet enhanced bacterial proliferation. In a complex host environment, several additional factors could contribute to this phenotype. First, *S. pneumoniae* infection often induces an early pro-inflammatory response that subsequently transitions into an immunosuppressive phase [19]. It is possible that reduced bacterial clearance due to immune exhaustion or macrophage dysfunction at later stages facilitates the persistence or regrowth of Δ*spuA* cells. Second, altered inflammatory milieu may create microenvironments with reduced antimicrobial pressure or increased availability of host-derived nutrients, both of which could favor bacterial survival and regrowth [20]. Third, differences in biofilm formation capacity might also play a role. Enhanced biofilm development can protect pneumococci from host immunity and antibiotics, allowing slower but more sustained bacterial proliferation [21]. Although SpuA is not directly implicated in biofilm formation, metabolic reprogramming caused by its deletion could indirectly influence extracellular matrix production or bacterial aggregation. Taken together, these alternative explanations suggest that the late-phase growth advantage of the Δ*spuA* strain likely arises from the interplay between metabolic adaptation, host immune modulation, and potential biofilm-related protection. Further studies combining immunological assays and biofilm quantification will be necessary to dissect these mechanisms in detail.

One plausible compensatory mechanism involves the increased utilization of amino acids. Transcriptomic data revealed upregulation of several tRNAs associated with amino acid transport and protein synthesis in the Δ*spuA* strain, suggesting a potential shift from carbohydrate-based metabolism to amino acid-based anabolism. Interestingly, SpuA deletion was also found to suppress the CcpA pathway in *S. pneumoniae*, a central regulator of carbon catabolite repression that coordinates carbohydrate uptake and utilization (Table S1). Since CcpA activity reflects the availability of glycolytic intermediates, its downregulation implies that the Δ*spuA* strain experiences limited access to preferred carbon sources and must rely on alternative metabolic routes to sustain growth [22]. In this context, the enhanced expression of amino acid–utilization genes may represent an adaptive response to maintain cellular energy and redox balance. Consistent with this idea, supplementation with cysteine markedly promoted the growth of Δ*spuA* bacteria in vitro, especially under formic acid stress. This observation suggests that cysteine can compensate for the metabolic imbalance caused by impaired glycolytic flux, thereby supporting bacterial survival and fitness under acid stress conditions.

In addition to its role as a metabolic byproduct, formic acid may also act as a metabolic signal that modulates bacterial physiology. Although no dedicated formic acid sensor or regulatory system has been identified in *S. pneumoniae*, evidence from other bacteria suggests that formate can function as a signaling molecule influencing gene expression and metabolic adaptation. For example, in *Escherichia coli*, formate acts as a signal in mixed-acid fermentation to regulate formate hydrogen lyase complex activity and redox balance [23]. Similarly, in *Listeria monocytogenes* and *Salmonella enterica*, formate has been reported to influence virulence gene expression and stress tolerance through transcriptional regulators responsive to metabolic acids [24,25]. These findings raise the intriguing possibility that *S. pneumoniae* may also sense formic acid indirectly through redox-sensitive regulators such as CcpA, thereby modulating tRNA expression and amino acid metabolism. However, the precise molecular mechanism underlying this potential signaling role remains unknown and represents an important avenue for future investigation.

It is important to note that our conclusions regarding amino acid utilization are currently based on indirect evidence, namely, tRNA expression profiles. While these data suggest enhanced amino acid involvement, the actual metabolic flux through specific amino acid pathways remains to be determined. To determine whether specific amino acids are preferentially catabolized for energy or biosynthesis, future studies should incorporate stable isotope tracing or targeted metabolomics.

Interestingly, the results challenge the conventional view of SpuA as a purely pro-colonization factor [6]. While SpuA facilitates early host cell attachment and nutrient acquisition, its absence may remove a regulatory check on bacterial proliferation, resulting in enhanced growth during later stages of infection. This suggests a more nuanced role for SpuA, potentially as a modulator of bacterial persistence and host–pathogen equilibrium. Understanding this dual functionality may have implications for vaccine design or therapeutic targeting, particularly in managing chronic or relapsing pneumococcal infections.

In conclusion, this study highlights the metabolic plasticity of *S. pneumoniae* in response to the loss of a key carbohydrate-processing enzyme. The observed shift toward acid resistance and amino acid utilization represents a novel adaptation strategy that enables survival in a nutrient-restricted, hostile lung environment. These findings broaden our understanding of pneumococcal pathophysiology and underscore the need for further investigation into the metabolic networks that govern bacterial fitness and virulence.

## Data Availability

The original contributions presented in this study are included in the article and Supplementary Materials. Further inquiries can be directed to the corresponding author.

## Supplementary Materials

The following supporting information can be downloaded at: https://www.mdpi.com/article/10.3390/microorganisms13102409/s1, Table S1: Differential expression of key metabolic regulatory genes in wild-type and Δ*spuA* strains under formic acid treatment.
