# Supplementary material for: SpuA-Mediated Glycogen Metabolism Modulates Acid Stress Adaptation via Formic Acid and Amino Acid Utilization in Streptococcus pneumoniae
Source: Microorganisms. 2025 Oct 21;13(10):2409. doi: 10.3390/microorganisms13102409 (PMC12565764; doi:10.3390/microorganisms13102409)
Supplement: Supplementary file 1 [file microorganisms-13-02409-s001.zip › microorganisms-3926645-supplementary.pdf]

Table S1. Differential expression of key metabolic regulatory genes in wild-type and Δ*spuA* strains under formic acid treatment

		**Δ*spuA* (without FA) vs. WT (without FA)**		**Δ*spuA* (without FA) vs. ΔspuA (with FA)**	
**Row-names**	Symbol	Fold Change	padj	Fold Change	padj
**SP_1999**	*ccpA*	-0.681674587	2.94E-05	0.396391688	0.027277539
**SP_1090**	*rex*	-0.304392593	0.107717726	-0.102216756	0.675528022
**SP_0730**	*spxB*	0.017040145	0.940101932	0.038975016	0.883863122
